# Pulsed Electric Field (PEF) treatment of progressive non-small cell lung cancer concurrently treated with immune checkpoint blockade: A case report

**DOI:** 10.1016/j.rmcr.2024.102018

**Published:** 2024-03-29

**Authors:** Marcelo Jimenez, Jose M. Fernandez, William S. Krimsky

**Affiliations:** a Salamanca University Hospital, Paseo de San Vicente, 58-182, 37007, Salamanca, Spain; b University of Salamanca, Paseo de San Vicente, 58-182, 37007, Salamanca, Spain; c Instituto de Investigación Biomédica de Salamanca (IBSAL) Paseo de San Vicente, 58-182, 37007, Salamanca, Spain; d Galvanize Therapeutics, Inc. 3200 Bridge Parkway Drive, Redwood City, CA, 94065, USA

**Keywords:** Pulsed electric field (PEF), NSCLC, Immune checkpoint blockade, Case report

## Abstract

Pulsed Electric Field (PEF) energy was delivered at the time of confirmational biopsy to ablate recurrent NSCLC in the right upper lobe (RUL) of the lung after recurrence while on durvalumab consolidation. The patient tolerated the procedure and exhibited stable disease at 6 and 12 months from time of durvalumab discontinuation and PEF treatment, respectively. This report represents the first use of the Aliya™ PEF system as a minimally invasive modality with potential to re-sensitize disease to immune checkpoint blockade (ICB) upon progression.

**Clinicaltrials.gov identifier:**

NCT04773275.

## 1 Introduction

The Aliya™ Pulsed Electric Field (PEF) system (Galvanize Therapeutics Inc., Redwood City, CA) received 510 (k) clearance by the FDA on June 17, 2022, for the surgical ablation of soft tissue [1]. PEF leads to cellular demise through local delivery of high-strength electric fields with mitigated impact to the surrounding stroma and critical structures [2]. It is postulated that PEF ablation of the tumor may induce remodeling of the TME and augment systemic therapies including ICB. Herein we present the first application of the Aliya PEF system in a patient with Stage IIIB non-small cell lung cancer (NSCLC) who progressed 6 months after durvalumab consolidation. Importantly, this case report highlights the promise of PEF delivery as a safe local ablative modality with the potential to augment ICB therapy.

## 2 Case presentation

**Fig. 1:**
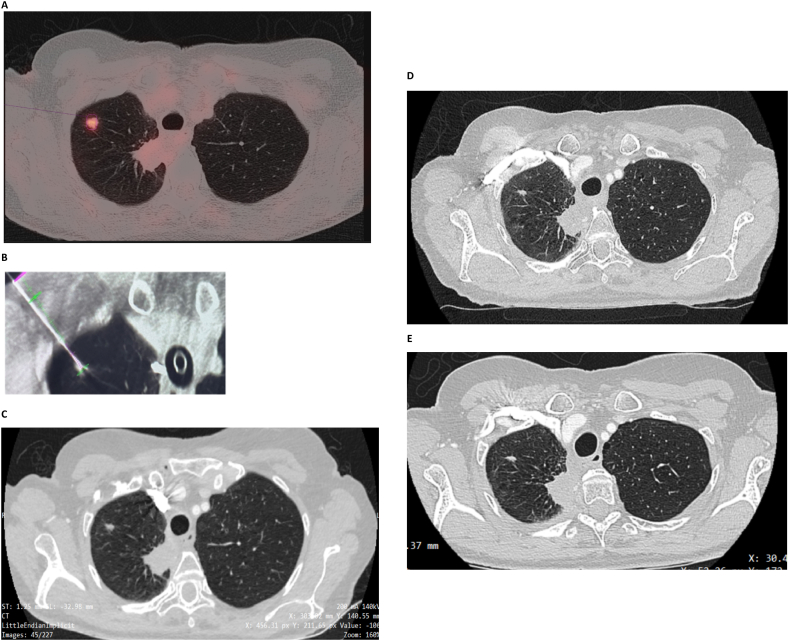
A) Axial PET-CT scan images of chest from March 2022 showing new 9 mm RUL and stable right paramediastinal disease; B) Percutaneous needle prior to delivery of PEF from June 2022; Follow-up images performed at 3-months (C) and 6-months (D), and 12 months (E).

In April 2021, a 66-year-old female originally presented with a right para-mediastinal mass (7.6 x 7 × 9.5 cm). Biopsy and molecular testing confirmed a Stage IIIB (cT4N2M0) NSCLC adenocarcinoma exhibiting PD-L1 > 1% in 50% of cells and negative for any driver mutations. The patient received four cycles of cisplatin and pemetrexed with concomitant radiotherapy (60 Gy) through July 2021. Consolidation with durvalumab began in September 2021 with a partial response noted after three months (2.8 x 3.1 × 6.3 cm). PET-CT imaging performed after 6 months identified a new 9 mm, FDG-avid (SUV max 5.5) nodule in right upper lobe (RUL) of the lung (Fig. 1A) consistent with early progression.

The patient consented to participate in a clinical study entitled “INCITE LS: A Clinical Evaluation of the Aliya™ System in Late-Stage Cancer” (NCT04773275). Briefly, INCITE LS evaluates patients who initially responded to ICB, but then develop progression. The primary outcomes included safety and radiologic local control. In June 2022, the patient underwent percutaneous Cone Beam CT-guided biopsy of the RUL lesion confirming recurrent disease. The lesion measured 10 × 9.5 mm and was located approximately 9 mm from the pleura. The percutaneous needle was positioned (Fig. 1B) and the first PEF dose was delivered to the distal portion of tumor and a second dose after retracting the needle 5 mm. This PEF system has a single dose setting which was designed to produce an approximately 1 cm^3^ ablation zone, and multiple doses can be stacked to cover larger areas. A pneumothorax was observed and determined by the treating physician to be related to the biopsy procedure. The patient was discharged home the next day and restarted on durvalumab.

In August 2022, 3-month imaging was performed per protocol. CT-scans of the chest revealed modest regression of RUL lesion (8 × 4 mm) with stable para-mediastinal disease (2.3 × 2.5 cm) (Fig. 1C). Six-month follow-up images in January 2023 were unchanged (Fig. 1D) and durvalumab was discontinued. Surveillance imaging at 12 months post-PEF treatment showed stable RUL (8 × 4 mm) and right paramediastinal (2.6 x 2.4 × 5.6 cm) disease and no new findings (Fig. 1E).

## 3 Discussion

### 3.1 Imaging discussion

Given the RECIST criteria, the imaging data from the Chest CT in the spring of 2022 after 6 months of treatment with Durvalumab, was consistent with disease progression. In the context of a differential diagnosis that includes granulomatous and/or infectious etiologies, an image guided biopsy was done prior to delivery of the PEF therapy confirming disease progression. Per protocol, a Chest CT was done in September 2022, or roughly 3 months after the index procedure, and demonstrated significant reduction in lesion volume compared to baseline (166 mm^3^ vs 200 mm^3^, respectively). In the context of the decrease in volume, the lesion was no longer thought to be amenable to repeat biopsy and suggested regression of disease. In January 2023, a six-month CT scan was done and confirmed disease stability. More specifically, there was no change to the index lesion when compared to the 3-month scan nor was there the interval development of any new foci of disease. As discussed, Durvalumab was discontinued at that same time, or roughly 6 months after the index procedure per country guidelines. A Chest CT at a total of 12 months from the procedural intervention and six months from discontinuation of the CPI therapy again confirmed disease stability, stable RUL (8 × 4 mm) and right paramediastinal focus (2.6 x 2.4 × 5.6 cm) and without new findings (Fig. 1E). Although no follow up PET-CT was conducted, prior work from a treat and resect trial demonstrated little impact on the underlying stromal elements of the treated areas [3]. Nevertheless, some residual scarring may be possible. In summary, imaging demonstrated that two doses of PEF treatment of early progressive Stage IIIB NSCLC to the RUL of lung resulted in stable disease at 12 months from post-PEF ablation.

### 3.2 Pathologic discussion

A biopsy of the new nodule was performed, and pathology confirmed recurrence of the NSCLC adenocarcinoma.

### 3.3 Brief review of literature

The PACIFIC trial was a randomized, placebo-controlled, phase 3 study evaluating durvalumab for up to 12 months in patients with stage III, unresectable NSCLC who did not progress after concurrent chemoradiotherapy [4]. In that study, the median progression-free survival was 16.9 vs 5.6 months (HR, 0.55; 95% CI, 0.45 to 0.68) and median overall survival 47.5 vs 29.1 months (HR, 0.72; 95% CI, 0.59 to 0.89) in favor of durvalumab [5]. The incidence of new lesions was 24.2% with 55% arising in the lung, while the median time to first subsequent anticancer therapy was 21.2 vs 10.4 months from randomization. Recently, PEF-energy has been shown to modulate the immune response in treatment naïve early-stage NSCLC with the induction of mature tertiary lymphoid structures [6].

### 3.4 Clinical discussion

In this case report, the patient developed early progression in the lung within 6 months from the start of durvalumab consolidation. Instead of considering a subsequent anti-cancer systemic therapy, the patient was enrolled in the INCITE-LS study and received a single PEF treatment (2 doses) for recurrent disease and remained on durvalumab for an additional 6 months. Currently, the patient has no evidence of new disease at 12 months post-PEF therapy and 6 months from the time durvalumab was discontinued. The observations reported herein raise the possibility that PEF therapy may have sensitized the tumor to the ongoing ICB similar to the effect that chemoradiation may have had on the primary tumor [7]. While encouraging, this preliminary observation requires prospective clinical validation. Currently, the role of PEF-energy is being studied for resectable Stage IIB-IIIA NSCLC (NCT05583188) in the neoadjuvant setting and for oligometastatic disease to the lung (NCT05890872) both in combination with systemic therapies.

## 4 Conclusion

• PEF energy was successfully delivered to a patient demonstrating progression on ICB for the first time.
• The therapy was delivered at the time of confirmational biopsy without any treatment-related adverse events.
• When considering the findings from the pivotal PACIFIC trial, this patient may have benefited from this minimally invasive, targeted intervention as compared to potential toxicities associated with subsequent anti-cancer therapies.

## Funding statement

Funding for the INCITE LS trial is provided to the Salamanca University Hospital by Galvanize Therapeutics, Inc.

## EC number and date of approval

C.E.I.M. reference 21/1648 (E.C·P·S.), approved February 7, 2022.

## Informed consent statement

The patient was identified as an appropriate candidate and consented for study, standard of care procedures, and publication of the data for the Ethics Committee-approved INCITE LS trial (NCT04773275).

## CRediT authorship contribution statement

**Marcelo Jimenez:** Data curation, Methodology, Writing – review & editing. **Jose M. Fernandez:** Writing – review & editing. **William S. Krimsky:** Conceptualization, Data curation, Writing – original draft.

## Declaration of competing interest

The authors declare the following financial interests/personal relationships which may be considered as potential competing interests:

William Krimsky is the CMO and an employee of Galvanize Therapeutics.

Marcelo Jimenez received support for travel from Galvanize Therapeutics and is a consultant for Medtronic and Intuitive Surgical.

Jose Fernandez declares no conflicts of interest.
